# User Testing of Information Materials Developed for the Australian National Lung Cancer Screening Program: A Qualitative Study

**DOI:** 10.1111/hex.70592

**Published:** 2026-02-10

**Authors:** Dan Luo, Kate L. A. Dunlop, Kathleen McFadden, Marianne Weber, Nicole M. Rankin, Claire Nightingale, Kate Broun, Hailey Fisher, Orelia Bello, Vivienne Milch, Rachael H. Dodd

**Affiliations:** ^1^ The Daffodil Centre The University of Sydney, and Cancer Council New South Wales Sydney Australia; ^2^ Faculty of Medicine and Health, Susan Wakil School of Nursing and Midwifery The University of Sydney Sydney Australia; ^3^ Faculty of Medicine and Health, School of Public Health The University of Sydney Sydney Australia; ^4^ Faculty of Medicine, Dentistry and Health Sciences, Melbourne School of Population and Global Health The University of Melbourne Melbourne Australia; ^5^ Prevention Division Cancer Council Victoria Melbourne Australia; ^6^ Lung Foundation Australia Brisbane Australia; ^7^ Cancer Australia Sydney Australia

**Keywords:** community participation, health communication, health workforce, lung cancer screening, patient information, qualitative research

## Abstract

**Introduction:**

The Australian National Lung Cancer Screening Program commenced in July 2025. This study aimed to explore the views of community members and the health workforce on the content, language, design and presentation of the draft information materials developed for the general population and health workforce.

**Methods:**

Semi‐structured interviews and focus groups were conducted between September and October 2024. Community members potentially eligible for the program and those interested in screening were recruited. Members of the health workforce who would be involved in screening were invited to participate. The teach‐back technique assessed community members comprehension of material content. An adapted user‐experience honeycomb model, along with a deductive thematic analysis approach, were used to identify aspects needing improvement.

**Results:**

A total of 25 community members and 44 health workforce participants took part. Shared and specific themes targeted for community and health workforces were identified. These included an *appreciation for clear language; content that is comprehensive, reliable, and transparent;* and *visually engaging design*. Specific themes focused on *enhancing the practical value of the materials for target audiences; clarifying and using professional terms and concepts;* and *adopting user‐friendly designs that accommodated the diverse needs of audience*. The role of family members in encouraging screening participation should be highlighted in the information materials. A standardised approach for assessing individuals smoking history in the program's eligibility criteria is warranted.

**Conclusion:**

These findings have guided the refinement of the draft informational materials developed for the general population and health workforce in the lung cancer screening program and could inform the development of materials for other cancer screening programs.

**Patient or Public Contribution:**

Community members and the health workforce were involved in the development of information materials.

## 1 Introduction

Lung cancer remains the most commonly diagnosed cancer and the leading cause of cancer‐related deaths worldwide [1, 2], presenting a significant global health challenge. In 2022, there were approximately 2.5 million new cases and 1.8 million deaths attributed to lung cancer globally [2, 3]. In Australia, approximately 15,122 new cases of lung cancer were diagnosed in 2024, with an estimated 8918 deaths from the disease [4].

Lung cancer screening using low‐dose computed tomography (LDCT) has emerged as a pivotal intervention for the early detection of lung cancer, demonstrating a significant reduction in mortality among high‐risk populations [5, 6]. Despite these benefits, participation rates in lung cancer screening vary across countries. In the United Kingdom, nearly 90% of individuals with a smoking history expressed interest in screening, with an uptake rate of 52.6% among those potentially eligible [7, 8]. Conversely, in the United States, participation remains consistently low (ranging from 5% to 16%) among eligible individuals, despite the screening program being in place for over a decade [9, 10]. Complex factors contributing to low uptake have been identified in previous studies, including lack of awareness about screening [11, 12, 13], low levels of health literacy [12, 14], demographic and socioeconomic factors (e.g., geographical remoteness, lack of health insurance) [15, 16, 17], psychological barriers (e.g., stigma associated with lung cancer, fear of diagnosis) [14, 17, 18], and healthcare system limitations (e.g., insufficient capacity to reduce scanning waiting times, inadequate resources for healthcare providers to refer patients to scans and facilitate timely treatment) [14].

In Australia, the National Lung Cancer Screening Program (NLCSP) commenced in July 2025 [19]. The program targets those at high‐risk of lung cancer, with eligibility criteria determined as those aged 50–70 years who are asymptomatic for lung cancer, have a smoking history of at least 30 pack‐years (e.g., smoking a pack of 20 cigarettes daily for 30 years), and are either currently smoking or have quit within the past 10 years [19]. A key focus of the NLCSP is to achieve equitable outcomes for priority populations, including Aboriginal and Torres Strait Islander peoples [20], individuals from culturally and linguistically diverse (CALD) communities, the LGBTIQA+ community, people living with disabilities, people living with a mental illness, and people living in rural and remote areas [21].

**Figure 1:**
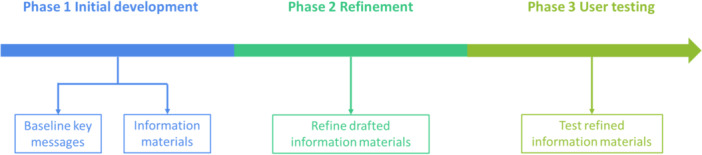
Three phases of the project *Design of information materials and education resources for the NLCSP*.

To increase awareness and knowledge of the NLCSP among community members and the health workforce, a suite of information materials and education resources was developed based on the team's previous work on lung cancer screening [13]. The development of these resources comprised of three phases (Figure 1). The aim of this current study was to conduct user testing of the information materials among community members and the health workforce, specifically aiming to explore views on the content, language, design and presentation of the information materials to inform the final stage of resource development.

## 2 Methods

### 2.1 Research Team and Reflexivity

The research team comprised 11 investigators. All researchers were female and experienced in qualitative methods and had expertise across behavioural science, public health, implementation research and cancer screening. The research team members have diverse cultural backgrounds, ranging from Australian and European to Asian. This diversity enhanced the interpretation of the study findings, as participants also represented varied cultural contexts.

### 2.2 Study Design

This study presents data for *Phase 3* of the project *Design of information materials and education resources for the NLCSP*. *Phase 1* involved the initial development of baseline key messages and content for information materials through workshops and a scoping review. *Phase 2* primarily focused on the content refinement of draft information materials for the community and health workforce through co‐design workshops with community members and health workforce and feedback from key stakeholders (i.e., the NLCSP's implementation partners, including Cancer Australia, the Department of Health, Disability and Ageing, and the National Aboriginal Community Controlled Health Organisation). *Phase 3* (the current study) centres on user testing of the refined information materials, guided by the Agency for Clinical Innovation (ACI) co‐design model Stage 3 (Understand) and Stage 4 (Improve), to inform final adjustments of the materials. The study findings were reported following the COnsolidated criteria for REporting Qualitative research (COREQ) checklist [22]. Details of the checklist are provided in Supporting File 1.

Semi‐structured interviews were conducted with community members, while focus groups and semi‐structured interviews were conducted with health workforce participants. The teach‐back technique was employed in the interviews with community members to evaluate their comprehension of the materials. This technique involves asking individuals to repeat the content in their own words to assess their understanding of the provided information [23]. It has been widely used in patient education contexts, such as supporting patient self‐management of chronic diseases [24, 25].

### 2.3 Participants

Eligible community participants included individuals potentially eligible for the NLCSP, and those who knew someone who might be potentially eligible for the NLCSP. Those potentially eligible for screening included individuals eligible now or in the future. Individuals who were unable to communicate in English were excluded. All members of the health workforce who would be involved in the NLCSP were eligible to participate, as lung cancer screening delivery involves a multidisciplinary approach. This included practising health providers, as well as those working in or with expertise in health management and health promotion.

### 2.4 Recruitment

A purposive sampling approach was used to recruit community participants in August and September 2024, aiming to include individuals from priority populations such as people living in rural and remote areas, CALD communities, and LGBTIQA+ communities. Potential participants from priority populations were purposively selected from the Lung Foundation Australia (LFA) participant database and the Join Us platform (https://www.joinus.org.au, an online database of community members interested in being contacted about research) and were invited to participate in the study. All interested individuals were included.

Purposive and snowball sampling approaches were employed to identify and recruit eligible health workforce from July to October 2024, aiming to cover a broad range of job roles and perspectives from professionals with varying levels of experience and expertise in lung cancer screening. Specific representation from primary care was prioritised in the recruitment process, given that recruitment of participants for the NLCSP will be led by this sector. Participants were identified through existing networks and connections of the project team, and by inviting them to share with others who may have been interested.

Potential participants were contacted by email, which included a link to an online expression of interest form with the Participant Information Sheet. Once the form was completed, participants were provided with the consent form, and an interview or focus group (health workforce only) was scheduled based on their availability.

### 2.5 Data Collection

Individual interviews and focus groups were conducted between September and October 2024. Information materials developed for community members (Supporting File 2) and the health workforce (Supporting File 3) were sent to the respective participants 1 week prior to the interviews and focus groups, allowing them to review the materials in advance. An interview guide (see Supporting File 4) was also developed by the research team.

Individual interviews with community participants were conducted by three researchers (D.L., K.D. and R.D.) via Zoom or telephone. This approach was chosen as the information materials developed for community members addressed sensitive topics (e.g., concerns about overdiagnosis and radiation exposure, and anxiety about screening results). Individual interviews therefore allowed participants to share their views openly without fear of being judged in a group setting. In contrast, focus groups with the health workforce were conducted via Zoom by five researchers (D.L., K.D., K.M., M.W. and R.D.), with individual interviews conducted when participants were unable to attend a focus group. Focus groups were used for the health workforce as they enabled the efficient collection of diverse perspectives from time‐poor participants and facilitated consensus on essential clinical information to be included.

During all sessions, each information material was either displayed on the screen or opened by the participants on their computer (for telephone interviews only). All participants were asked to share their views and feedback on the content, language, design and what they liked or disliked about the resource. Community participants were also asked to repeat parts of the resource back in their own words (i.e., the teach‐back technique). After interviewing 25 community participants and 44 healthcare workers, no new feedback was identified, indicating that data saturation was achieved.

All sessions lasted between 30 min and 1 h and were audio recorded. Each participant was offered an e‐gift voucher (each community member: $50; each health workforce: $100) as a token of appreciation for their time and participation. The interview and focus group recordings were transcribed verbatim using Microsoft Word by four researchers (D.L., K.D., K.M. and R.D.), with all identifiable information removed from the transcripts.

### 2.6 Data Analysis

All transcripts were quality checked by three researchers (D.L., K.D. and K.M.) before data analysis. Data analysis was conducted in three steps. In step one, two Excel spreadsheets were created to extract relevant responses to each resource developed for community members and the health workforce respectively. Community participants' responses were independently extracted and coded by two researchers (D.L. and K.D.) and discussed to reach an agreement. All health workforce participants' responses were extracted and coded by one researcher (K.M.) and reviewed by another researcher (R.D.) to ensure accuracy.

In step two, the adapted user‐experience honeycomb model [26] was employed to guide categorisation of relevant responses. This model was chosen as it is used to evaluate healthcare‐related information materials from users' perspectives and experiences, aligning with the aim of our study. The adapted model evaluates information materials from eight aspects: understandability, usefulness, usability, desirability, accessibility, credibility, affiliation and findability [26]. Brief descriptions of each aspect are presented in Table 1. A researcher (D.L.) initially used the adapted model to group relevant coded responses into the eight aspects or categories, discussed the grouped results with another researcher (K.D.), and made adjustments across categories to ensure consistent interpretation of the findings. An example that demonstrates the process of using the adapted model to guide grouping of relevant coded responses into the eight categories of the model is presented in Table 2.

**Table 1:** Brief descriptions of each aspect of the adapted user experience model [26].

Category	Description
Understandability	The degree to which the information materials are clear and easy to comprehend.
Usefulness	The degree to which the information materials are useful or valuable to users.
Usability	The degree to which the information materials are easy to use and satisfying.
Desirability	The degree to which the information materials are desired or appealing to users. It also reflected their emotional responses.
Accessibility	The degree of ease with which all users can access and use the information materials.
Credibility	The degree to which the information materials are trustworthy and reliable.
Affiliation	The degree to which users feel a sense of connection with the information materials.
Findability	The degree of ease with which users can locate the information materials they need.

**Table 2:** Example of using the adapted model to guide group of coded responses into each category of the model.

Category of the model	Example of coded response	Code
Understandability	1.1. I thought overall they were very clear and easy to understand. (CM‐4)^a^ 1.2. I like the document, thought it was clear. (HW‐FG‐1)^b^ 1.3. The last one is a bit confusing. Have a history of 30 pack years. Do they mean 30 a day or a pack? (CM‐3) 1.4. An authorised medical practitioner. Other times you're talking about GPS. So who's an authorised medical practitioner? (HW‐FG‐1)	1.1 and 1.2: Information is clear; Information is easy to understand 1.3: Need for clarification of 30 pack years 1.4: Need for concise use of professional terms
Usefulness	2.1. I've found it very comprehensive reading the whole package. (CM‐17) 2.2. Comprehensive, salient points covered…for a new program [Full resource for GPs] is useful…(HW‐FG‐3) 2.3. And then I think there needs to be a greater emphasis on encouraging, like getting younger people to encourage their older family members to get screening. Yeah. I feel like that's a huge issue…It's not emphasised enough in these documents I feel…Yeah, I think it should be included in the FAQ as well to encourage younger people to get their older family members to get screening done. (CM‐13) 2.4. [Need] calculator for pack [year]. (HW‐FG‐6)	2.1 and 2.2: Information is comprehensive 2.3: Emphasise family members role in facilitating screening 2.4: Need smoking history calculator
Usability	3.1 It [lung cancer symptoms] should be actually probably in bold letters… (CM‐5) 3.2 …lots of information, need to read many times… Font too small, text heavy, can be shortened (HW‐FG‐3)	3.1 Highlight lung cancer symptom for community members 3.2 Excessive information; not easy to read for health workforce
Desirability	4.1 …these are nice coloured pictures and they look welcoming. You know, that something you want to look at. (CM‐2) 4.2 [Conversation starters] Looks good, eye catching (HW‐I‐12)^c^	4.1 and 4.2: The information material is engaging; the information material is attractive.
Accessibility	5.1 I think it's important to have it [the information] in the audio form. (CM‐4) 5.2 [Add] clickable table of contents for online version. (HW‐FG‐1) 5.3 Also add for more information in other languages, please go to the website or ring TIS on number. (CM‐20) 5.4 …I assume this would be available in other languages for those for whom English is not their first language? (CM‐4)	5.1 and 5.2 Need audio format to deliver information; Add online format to deliver information 5.3 and 5.4 Need for multilingual information materials
Credibility	6.1 I really like the logo, national lung cancer screening programme…, I think it's looks really good. (CM‐6) 6.2 I quite liked it [logo]. (HW‐FG‐1)	6.1 and 6.2 Appreciation of the logo
Affiliation	7.1 …as somebody who has had a lung nodule diagnosed many years ago…it became pertinent to me and relevant. (CM‐4) 7.2 It's about making sure that the right people see the right resources. Or what information is for whom in this [material]. (HW‐I‐13)	7.1 Information is pertinent to individual's health condition 7.2 The information lacks specificity for different professional roles
Findability	N/A	N/A

^a^ CM‐X refers to community member X.

^b^ HW‐FG‐X refers to health workforce' focus group X.

^c^ HW‐I‐X refers to health workforce ‘individual interview X.

In step three, deductive thematic analysis was conducted by one researcher (D.L.) to explore theme within each category of the adapted model. This analysis approach was selected as it aligns with Braun and Clarke's recommendation when a pre‐existing theory or framework is used to inform theme development [27]. The category ‘findability’ was not included in our analysis since each information material was directly presented to the participants during the interviews. The codes were sorted into potential themes, which were then iteratively refined through regular discussions among the research team to ensure that the final themes were coherent and accurately reflected the data. An example that demonstrates the theme development and refinement process is presented in Table 3. Each of the resources for the community members underwent readability analysis (D.L. and K.D.) using the SHeLL Editor (https://shell.techlab.works/) to ensure that each resource met an acceptable reading level of grade 6 of Australian educational system standard.

**Table 3:** Example of theme development and refinement process through deductive analysis.

Category of the model	Example of refined theme	Example of potential theme	Code example
Understandability	*Shared theme*: Language found to be clear and easily understandable	*Shared theme*: Information is clear and easily understandable	1. Information is clear 2. Information is easy to understand
	*Community specific theme*: Need for clarification of some professional terms and concepts	*Community specific theme:* Need for clarification of certain concepts	1. Need for clarification of 30 pack years; 2. Need for clarification of natural radiation
	*Health workforce specific theme:* Need for concise use of medical professional nomenclature	*Health workforce specific theme:* Need for concise use of professional terms and concepts	1. Need for concise use of professional terms; 2. Interchangeably use of concepts (e.g., cigarettes vs. tobacco)
Usefulness	*Shared theme*: Information found to be comprehensive	*Shared theme*: The information is comprehensive	1. Information is comprehensive; 2. Information covered everything
	*Community specific theme:* Enhancement of practical value for the community	*Community specific theme:* Enhance practical value of the information materials designed for community	1. Emphasise family members role in facilitating screening
	*Health workforce specific theme:* Enhancement of practical value for the health workforce	*Health workforce specific theme:* Enhance practical value of the information materials designed for health workforce	1. Need smoking history calculator; 2. Need more explanations or scripts about the program ineligibility
Usability	*Community specific theme:* Adoption of community‐friendly design	*Community specific theme:* Use community‐friendly design	1. Highlight lung cancer symptom for community members; 2. Appreciate the non‐alarmist approach to present nodule management protocol
	*Health workforce specific theme:* Adoption of health workforce‐friendly design	*Health workforce specific theme:* Use health workforce‐friendly design	1. Not easy to read for health workforce; 2. Excessive information
Desirability	*Shared theme*: Appreciation of visually engaging design	*Shared theme*: Visually engaging design	1. The information material is engaging; 2. The information material is attractive
Accessibility	*Shared theme:* Need for diverse information access channels	*Shared theme:* Need for various information access avenues	1. Need audio format to deliver information; 2. Add online format to deliver information
	*Community specific theme:* Need for language inclusivity	*Community specific theme:* Need for language diversity in information materials	1. Need for multilingual information materials
Credibility	*Shared theme*: Appreciation of authoritative branding and logo	*Shared theme:* Use authoritative branding and logo	1. Appreciation of the logo; 2. ‘Australian Government’ branding is reliable
Affiliation	*Community specific theme:* Value information pertinent to personal health experiences	*Community specific theme:* Value information pertinent to personal health condition	1. Information is pertinent to individual's health condition
Findability	N/A	N/A	N/A

### 2.7 Trustworthiness

Credibility and confirmability of the findings were enhanced through the use of an investigator triangulation strategy, which involves employing two or more researchers in the data collection and analysis stages [28, 29]. Confirmability was achieved by providing a detailed description of the study methods and maintaining an audit trail for data collection and analysis [30]. The transferability was enhanced by providing sufficient information on the research context and participants' characteristics to allow readers to assess whether the findings could be applied to other settings.

### 2.8 Ethics

This study was approved by The University of Sydney Human Research Ethics Committee (2024/HE000700).

## 3 Results

A total of 69 individuals participated between September and October 2024, including community members (*n* = 25) and health workforce (*n* = 44). Of the community participants, 12 individuals met the eligibility criteria for the NLCSP. Most community participants were female (*n* = 19), resided in Victoria (*n* = 10), and had a history of smoking (*n* = 12). Among the eight community participants with a cultural background, three had a Chinese cultural background. For the health workforce participants, 28 individuals took part in seven focus groups, 15 participated in individual interviews, and one provided written feedback due to unavailability. Most health workforce participants were general practitioners (GPs) (*n* = 21) who practised in New South Wales (NSW) (*n* = 19). The detailed characteristics of community and health workforce participants are presented in Table 4.

**Table 4:** Characteristics of the community and health workforce participants.

Characteristics	Participants number
**Community members (*n*** = **25)**	
Age	60.4 ± 14^a^
Gender	
Female	19
Male	6
Residential state	
Victoria	10
New South Wales	5
Queensland	5
Western Australia	4
South Australia	1
Priority population^b^	
Live in rural and remote area	12
LGBTIQ+ community	4
Living with mental illness	5
Ethnic background	
Chinese	3
Croatian	1
Egyptian	1
Greek	1
Italian	1
Macedonian	1
Smoking status	
Person who used to smoke	12
Person who has never smoked	7
Person who smokes	6
**Health workforce (*n*** = **44)**	
Professional role	
General practitioner	21
Health promotion, screening or smoking cessation manager/expert	11
Respiratory physician	3
Radiographer	2
Lung cancer or oncology nurse	2
Radiologist	1
Thoracic surgeon	1
Medical oncologist	1
Pharmacist	1
Primary care practice manager	1
State of practice	
New South Wales	19
Victoria	9
Western Australia	6
Australian Capital Territory	4
Queensland	3
Tasmania	2
Northern Territory	1

^a^ Data presented as mean years and standard deviation.

^b^ Some participants hold multiple characteristics (e.g., living in rural and remote areas and having mental illness).

**Figure 2:**
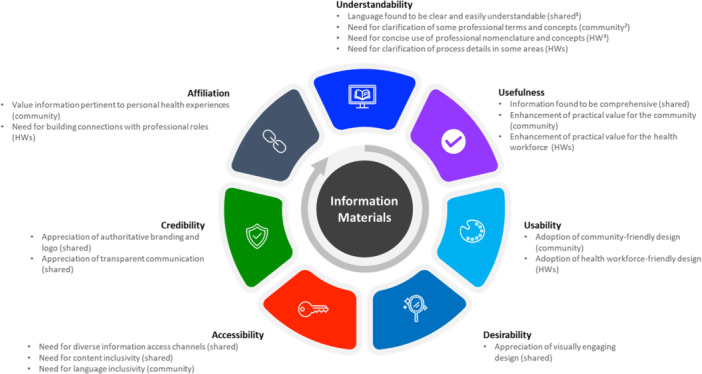
Summary of shared and distinct themes within each category. *Note:* ^1^shared = shared theme between community members and healthcare professionals; ^2^community = specific theme for community members; ^3^HWs = specific theme for health workforces.

Overall, both community participants (including those eligible and ineligible for screening) and health workforce participants provided positive feedback about the information materials. They perceived the majority of the information to be clear, comprehensive, and well‐designed, which aligned with the readability analysis showing that all community information materials met a grade 6 reading level. Suggestions for amendments, such as clarification of factual information (e.g., pack‐year, meaning of low dose in the LDCT), emphasis in some key areas (e.g., encouraging younger people to speak with older family members about screening), and wording changes (e.g., ‘smoke cigarette’ to ‘smoke tobacco cigarettes’), varied between the two groups of participants. A summary of themes (including shared and distinct themes between community and health workforce participants) identified within each category of the adapted user‐experience honeycomb model is presented in Figure 2. All illustrative quotes alongside participant's identifiers are outlined in Supporting File 5 to support each theme.

The iterative data analysis process revealed that different aspects of the user experience were interrelated. For instance, the use of visual elements (e.g., pictures, images, figures, colours) not only enhanced the attractiveness of the informational materials (desirability) but also facilitated the audience's understanding of complex information by presenting it in a straightforward manner (usability).

### 3.1 Understandability

#### 3.1.1 Language Found to Be Clear and Easily Understandable (Shared Theme)

Both community and health workforce participants consistently agreed that most of the language used in the information materials was clear, and easy to understand. Community participants particularly appreciated the use of simple language, which made the information self‐explanatory and accessible to a broad audience. Several also praised the well‐structured content, noting that it provided a clear and effective guide for individuals participating in the NLCSP. Similarly, health workforce participants also found the materials effectively conveyed key messages, making them easy to comprehend.

#### 3.1.2 Need for Clarification of Some Professional Terms and Concepts (Community Specific Theme)

Although most information was considered clear, community participants still expressed the need for clarification of certain professional terms and concepts related to smoking history and radiation exposure in the context of lung cancer screening. During the interviews, a number of community participants mentioned the term ‘pack‐years’ was confusing. Some were concerned that this concept might not be widely understood, while others sensed difficulty in calculating ‘30 pack‐years’ themselves, especially for those who also smoke substances (e.g., cannabis) other than tobacco cigarettes. Revision to the materials included providing specific examples to calculate 30 pack‐years (e.g., a pack a day for 30 years, 2 packs a day for 15 years). One participant also raised concerns about the accuracy of assessing smoking history, noting that individuals may underreport the amount they smoke to the GP.

Several community participants also stated that they were not familiar with radiation exposure during CT scans, hence requesting further explanation about what low‐dose means and the impacts of low‐dose radiation on health. Revision to the materials included clarifying radiation of low‐dose CT scans and emphasising its safety on health. Despite the concept of ‘natural radiation' being used to assist in explaining the small amount of radiation, it caused another point of confusion associated with its meaning and sources. Community participants suggested that providing some examples of natural radiation sources, such as solar radiation, and mobile phones, could enhance their comprehension and reassurance. These examples were added in the materials.

#### 3.1.3 Need for Concise Use of Professional Nomenclature and Concepts (Health Workforce Specific Theme)

In addition to emphasising the importance of clear and accessible language, the health workforce participants highlighted the importance of using precise professional terminology and concepts across all information materials. For instance, most health workforce participants highlighted that interchangeable use of ‘tobacco’ and ‘cigarettes’ in the eligibility criteria caused confusion. Since there were different forms of tobacco use, such as rollies, cigars, pipes, tobacco cigarettes, chew or sniff tobacco, shisha, and other tobacco products, they suggested clearly distinguishing various forms of tobacco consumption while describing individuals' smoking history. This would help potential participants to better understand the eligibility criteria. Revisions to the material included consistently using ‘tobacco cigarettes’ in the eligibility criteria. The health workforce participants also noted that the ambiguous use of some professional titles, such as ‘other medical practitioners’, led to uncertainty about roles and responsibilities and who was being referenced. To avoid confusion, it was replaced with ‘requesting practitioners’ in the materials. Several health workforce participants also questioned the use of *‘*self‐referral for a CT scan’ to describe individuals who seek to participate in the NLCSP, since eligible participants need to obtain a low‐dose CT scan request form from a GP or eligible health practitioner. Health workforce participants suggested replacing it with ‘self‐identify for the program’ for clarity. A corresponding change was also made to the materials.

#### 3.1.4 Need for Clarification of Process Details in Some Areas (Health Workforce Specific Theme)

The health workforce participants also asked some details about the NLCSP design which required clarification in the program guidelines before they were updated across the information materials. For instance, some requested more details about who (i.e., clinics or individual medical specialists) have the authority to register participants on the National Cancer Screening Register (NCSR), how to determine whether a participant remains enrolled in the NLCSP if they change practices, and which radiology providers can the requesting practitioners send participants to. Several also asked about the division of responsibility for participant recall of investigation and details of follow‐up scans, including who calls participants, who is responsible for follow up scans, and whether follow‐up scans referred exclusively to LDCT scans or other type of scans. These details were subsequently added to the materials.

### 3.2 Usefulness

#### 3.2.1 Information Found to Be Comprehensive (Shared Theme)

Both community and health workforce participants considered the information materials to be comprehensive and useful. Community members appreciated that the materials were highly informative and covered a broad range of topics, particularly the aim of the NLCSP, key considerations for before, during and after the scan, potential concerns about screening, and steps to take if an individual is not eligible. They described the information as a step‐through process, providing a clear guide to the screening journey and what to expect.

Health workforce participants found that the ‘Full resource for GPs’, ‘Key evidence for the NLCSP’, and ‘Frequently Asked Questions (FAQs)’ materials were particularly useful in understanding the scope of the program and providing guidance to patients. Several health workforce participants also pointed out areas where the information could be refined, such as combining similar information in the ‘FAQs’ material about why healthcare professionals should encourage their patients to screen for lung cancer.

#### 3.2.2 Enhancement of Practical Value for the Community (Community Specific Theme)

Community and health workforce participants highlighted the need to further enhance the practical value of the information materials tailored to each group. Community members, particularly those from CALD communities, suggested placing a greater emphasis on encouraging younger people to speak with their older family members about screening. They noticed that this aspect was not sufficiently emphasised across all materials and suggested incorporating it more explicitly into those that did not address it, such as the ‘FAQs About the Program’ and the ‘Storyboard of Scanning Video’. Corresponding changes were made to the materials based on these suggestions.

#### 3.2.3 Enhancement of Practical Value for the Health Workforce (Health Workforce Specific Theme)

The health workforce participants, on the other hand, focused more on the available tools and resources that could further improve practice, in particular the process in clinical settings. For instance, some health workforce participants expressed a need for a pack‐year calculator and suggested adding relevant information that could direct requesting practitioners to the calculator. Some requested guidance on converting measures of smoking exposure from other forms of tobacco use (e.g., grams per week for rolled cigarettes) into pack‐years. Several health workforce participants requested more explanations or ready‐made scripts about the ineligibility for the program for the ease of providing it to the ineligible individuals. They also suggested adding some strategies for communicating with ineligible participants. A detailed explanation of ineligibility for the program, along with communication strategies, was added to the materials.

### 3.3 Usability

Both groups valued information materials that were visually clear, well‐structured, and engaging, and viewed these as essential elements for effectively conveying extensive and complex messages.

#### 3.3.1 Adoption of Community‐Friendly Design (Community Specific Theme)

Community members appreciated visual designs that simplified complex information. For instance, most provided positive feedback on the presentation of the nodule management protocol, appreciating its gradual and non‐alarmist approach. They also praised the ‘Eligibility Criteria’ figure since it condensed complex information and presented it in a clear and straightforward manner, which made it easy to read and understand. Several community participants also appreciated the use of visual aid designs, such as colour (i.e., colour‐coded graphs, headings), icons, and tick boxes, noting that it enhanced their engagement and comprehension of the information as presented in digestible bites. Community members also emphasised the importance of identifying critical symptoms of lung cancer and suggested highlighting this information in bold or bigger font to ensure visibility. The symptoms of lung cancer were bolded in response to this feedback.

#### 3.3.2 Adoption of Health Workforce‐Friendly Design (Health Workforce Specific Theme)

Health workforce participants appreciated the succinct design, preferably one‐ to two‐page documents, to facilitate quick reference in fast‐paced clinical settings. Although they acknowledged that most information materials developed were helpful and appreciated the use of visual design such as colour and checklists, they perceived excessive information to make a resource appear overwhelming and not easy to read, particularly when presented in small font. To improve usability, they suggested shortening the lengthy text in materials and incorporating more visual elements, such as tables and graphs to highlight key information. Corresponding changes were made, including incorporating key information (e.g., screening results, resources available to health providers) into tables, and using a checklist to present key items when enrolling participants into the program.

### 3.4 Desirability

***Appreciation of visually engaging design (shared theme)*** shaped participants perceived desirability of the information materials. Both groups of participants highlighted that visually engaging designs could effectively capture users' attention. Community members particularly appreciated the use of colour images featuring real people, noting that these images appeared welcoming, pleasing to the eye, and both reassuring and comforting, while also humanising the content and motivating users to want to go for screening. They further praised the lung‐shaped logo with its approachable tones, and suggested that a thoughtfully designed brochure cover, using colours to sell the message (e.g., bright yellow) or evoke associations with medical services (e.g., calm blue), could serve as an effective hook to encourage further engagement with the materials. Health workforce participants also remarked on the effectiveness of certain design styles, such as the eye‐catching ‘Conversation Starters’, in capturing their attention.

### 3.5 Accessibility

#### 3.5.1 Need for Diverse Information Access Channels (Shared Theme)

To enhance accessibility, both participant groups proposed diverse delivery formats, such as audio format for the blind and low vision community, and various print and digital versions, to meet different communication needs. Community members preferred a mix of A4 and trifold print formats, while the health workforce favoured portrait A4. Community participants also recommended replacing directional arrows in print materials to avoid confusion. Digitally, the health workforce suggested adding more interactive elements such as a clickable table of contents to enhance navigation. Corresponding revisions were made to the online version of the health workforce materials. All community members also appreciated the ‘Storyboard of Scanning Video’ and regarded the combination of audio and text as an effective way to keep content engaging and easier to understand.

#### 3.5.2 Need for Content Inclusivity (Shared Theme)

Both community and health workforce participants emphasised the need for more inclusive content in the materials. Community members were concerned about the lack of cultural diversity, which could alienate diverse populations. Suggested solutions included replacing some images, adding information about interpreter services and a link to multilingual resources, and disability accommodations, indicating whether there are special arrangements available during screening for people who live with disabilities. Similarly, health workforce participants recommended incorporating culturally informed perspectives, such as the meanings attributed to smoking varies across cultures (e.g. social norm, gesture of hospitality), to enhance comprehension and facilitate more effective communication. Revisions to the materials included using images of people from different cultural backgrounds, adding information on additional support services for priority populations (e.g., interpreter services, support for indigenous people and those living with disabilities), and including information on considering participants' cultural perspectives.

#### 3.5.3 Need for Language Inclusivity (Community Specific Theme)

Community members also highlighted the need for using inclusive language in the information materials. While most appreciated the use of plain language, which made the materials broadly accessible, some participants recommended providing multilingual materials to better support individuals who do not speak English. The final versions of the English materials were also translated into multiple languages (e.g., Arabic, Chinese and Vietnamese).

### 3.6 Credibility

#### 3.6.1 Appreciation of Authoritative Branding and Logo (Shared Theme)

Both groups of participants perceived the information materials as trustworthy and reliable due to the ‘Australian Government’ branding. They particularly appreciated the lung‐shaped logo, and appealing blue and green colour palette associated with the program, which distinguished the materials from the more austere government communications. However, several health workforce participants noted that the logo and colour palette closely resembled those of National Asthma Australia, raising concerns about a potential mix‐up between the two entities.

#### 3.6.2 Appreciation of Transparent Communication (Shared Theme)

Several participants also expressed that transparent communication was a cornerstone of credible information materials. Community and health workforce participants appreciated that the informational materials were honest about key facts regarding lung cancer and perceived that information transparency was essential for building trust among the audience.

### 3.7 Affiliation

#### 3.7.1 Value Information Pertinent to Personal Health Experiences (Community Specific Theme)

Community members perceived that information pertinent to their previous health experiences, such as a lung nodule diagnosis, was particularly important compared to other points of information. This information not only enhanced their understanding of lung health but also potentially reduced their fear and concerns of the screening results.

#### 3.7.2 Need for Building Connections With Professional Roles (Health Workforce Specific Theme)

Health workforce participants noted that specifying who the information is for, and what the document is about, would help them quickly identify relevant resources and navigate the content more efficiently in alignment with their professional roles and needs. For example, several participants were uncertain about the target audience for ‘FAQs’ material (i.e., developed for all health workforce, or for practitioners only). This information (i.e., requesting practitioners) was added to the relevant materials.

## 4 Discussion

**Figure 3:**
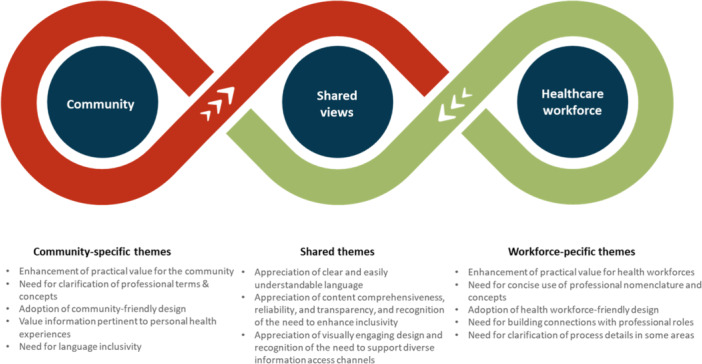
Summary of shared and community‐ and healthcare workforce‐specific themes.

This study examined the views of community members and the health workforce on the content, language, design and presentation of the draft information materials developed for the Australian NLCSP. We identified shared and specific themes targeted for community and health workforce audiences (see Figure 3). The shared themes include *appreciation of clear and easily understandable language*; *appreciation of content comprehensiveness, reliability, and transparency alongside recognition of the need to enhance inclusivity*; and *appreciation of visually engaging design and acknowledgement of the need to support diverse information access channels*. The specific themes primarily focus on *enhancing the practical value for target audiences*; *clarifying and accurately using professional terms and concepts*; and *adopting user‐friendly designs* that accommodate the diverse needs of different audiences. Areas were also highlighted the use of pack‐years as a criterion for selecting high‐risk individuals for the NLCSP might need to be evaluated after the initial program implementation. Based on our findings, the research team further revised the information materials to ensure that the final versions meet the information needs of both groups (available at https://www.health.gov.au/our-work/nlcsp/resources).

### 4.1 Shared Themes

Our findings highlight the importance of clear, easily understandable language in both sets of information materials. This aligns with the suggestions provided by the Australian Human Rights Commission for written communications [31]. In line with previous research, our analysis also revealed the importance of ensuring that information materials are comprehensive, reliable, transparent and inclusive. A systematic review that summarised the current state of lung cancer screening programs worldwide emphasised the need to cover key content for lung cancer screening (e.g., the benefits, and risks of screening) in the educational resources designed for the general public [32]. Furthermore, empirical studies indicate that information reliability is ‘the most important thing’ for audiences [33], and the use of positive design cues, such as logos of trusted organisations, can enhance the perceived credibility of the message [34]. Maintaining transparency and honesty of the information, particularly regarding potential harms of lung cancer screening, is also regarded to be of the same importance [35]. Participants in our study similarly suggested enhancing the inclusivity of the information materials. This aligns with findings from a literature review highlighting that incorporating culturally appropriate information into educational materials is essential for facilitating awareness of lung cancer screening [36].

This study found that using visually engaging designs, for example, colourful images featuring real people and an eye‐pleasing logo with approachable tones, can effectively capture audience attention. This echoes the findings of research on the impact of visual design on user engagement with health information. For instance, one study analysing the visual design of 94 nutritional websites found that human imagery significantly influenced viewers first impressions and could make them more receptive to the information presented [37]. The resources developed to mitigate lung cancer stigma also recommend using colour judiciously in written content to achieve the desired effect. For example, bright colours such as green and blue capture attention and evoke positive emotions more effectively than black and red [38]. Our analysis also revealed that a visually engaging design could enhance the usability of information materials and promote cultural diversity. This aligns with principles of effective information visualisation that improve user experience and inclusivity [39].

### 4.2 Community‐Specific Themes

The role of family in motivating family members to participate in screening was a key finding, aligning with a recent study that recommended family members involvement as a potentially valuable enabler for increasing lung cancer screening uptake [40]. The influential role of family support in motivating individuals to undergo screening has also been observed in other cancer screening programs [41].

Clarifying professional terms and concepts for community members, particularly the term ‘pack‐years’, was also highlighted. Although the NLCSP encourages community members to determine their pack‐years with the support of a GP or non‐GP specialist, concerns about potential consultation costs may deter participation [42]. Additionally, a recent American study found that a smoking duration cutoff (e.g., 20 years) appeared more precise than a pack‐year cutoff (e.g., a 20‐pack‐year history) for assessing smoking history [43]. Evaluation of the use of pack‐years as an appropriate criterion for selecting high‐risk individuals for the NLCSP in Australia will be necessary as the program is implemented. Such research may inform potential adjustments to the NLCSP eligibility criteria in the future. The updated evaluation approach for smoking history should also be explained in detail in the updated information materials to ensure it is easily understood by the public.

Utilising community‐friendly designs featuring infographics and various visual aids was also acknowledged by participants. The role of infographics and visual aids in improving comprehension and engagement has also been explored in previous research. A study comparing knowledge acquisition from infographics versus plain language summaries found that infographics were considered more understandable and reader‐friendly [44], as they not only capture users' attention but also help convey key messages effectively [45, 46]. Similarly, visual aids not only complement textual content and enhance the overall appeal of information but have also been found to improve audience comprehension and risk literacy and make health information more accessible [47, 48].

### 4.3 Health Workforce‐Specific Themes

Practical tools and resources suggested as necessary for the healthcare workforce included a pack‐year calculator and methods for converting other forms of tobacco exposure into pack‐years. To date, various pack‐year calculators have been developed, including those that account for alternative forms of tobacco use (e.g., cigars, pipes, loose tobacco, and rollies). However, no standardised method has been established for a comprehensive and accurate assessment of an individual's smoking history. Future research could focus on the development of a standardised pack‐year assessment tool or refining existing tools to improve accuracy and consistency.

Precise professional terminology and concepts were also highlighted by health workforce participants as important. In our attempts to simplify resources to ensure accessible information and promote efficiency in practice, we failed to provide adequate, precise, and consistent professional terminology and concepts throughout the resources. The juxtaposition of these approaches highlights the challenge in translating technical language and concepts into plain English.

The health workforce preferred a succinct design with enhanced visual elements (e.g., tables, graphs, and images) to present key messages to enable clinicians to quickly locate essential information. Previous research found that visualising information could potentially reduce cognitive load [49], a concept referring to the mental effort required to process and retain new information [50], thereby promoting more effective learning [51]. Additionally, the application of cognitive load theory in modern educational practices has also demonstrated the value of information visualisation in improving effective learning [51].

### 4.4 Strengths and Limitations

The strengths of the study include the diverse range of participants from the community (including representatives from priority populations though the numbers were small) and the health workforce, which allowed for various perspectives to be captured. Additionally, the use of an investigator triangulation strategy in interviews with both groups, along with the teach‐back technique to assess community members' comprehension of the content, further enhanced the credibility of the study findings. Another novel aspect of this study was the application of an adapted user‐experience honeycomb model to guide a comprehensive analysis of the data. This approach uncovered key findings related to the general and specific principles of designing information materials tailored to different audiences.

Limitations of this study include the lack of targeted consultations for priority populations. Although clear themes around diversity and accessibility were found in our study, future studies should engage with priority populations to understand their specific preferences and views, ensuring that the general information materials developed in this research project are adapted to meet their unique information needs. As the information materials were developed in English, another limitation of this study was that people who do not speak English were excluded from this study. These materials were reviewed further by CALD participants as part of another project conducted with LFA. Additionally, the materials co‐designed with community members were also found to lack cultural diversity since the number of co‐design workshop participants from culturally diverse backgrounds was low (< 25%). Further research should consider involving more individuals from CALD communities, including those who do not speak English, to enhance the cultural diversity of the materials. The study findings also have limited generalisability where certain aspects may only be applicable to the Australian setting and/or lung cancer screening specifically. Another limitation is that community participants were recruited from two sources (i.e., the LFA database and the Join Us platform), which may not represent all eligible screening individuals. Only those who expressed interest by responding to the online advertisements from LFA and Join Us were invited to participate in the interviews. This recruitment approach may have overlooked eligible screening individuals who were interested in the study but had limited internet access. Future studies should consider employing more diverse recruitment avenues to capture a broader range of views from individuals with varying demographic characteristics.

## 5 Conclusion

This study identified both general and specific themes for designing information materials targeting both community members and the health workforce for the Australian NLCSP. These findings have the potential to guide the development or adaptation of information materials for lung cancer screening programs in other settings or groups (e.g., priority populations) and highlight potential areas where interventions could increase participation.

## Author Contributions

**Dan Luo:** methodology, formal analysis, investigation, data curation, writing – original draft, visualization, project administration, writing – review and editing. **Kate L.A. Dunlop:** methodology, formal analysis, investigation, data curation, writing – original draft, project administration, writing – review and editing. **Kathleen McFadden:** formal analysis, investigation, data curation, writing – review and editing. **Marianne Weber:** investigation, writing – review and editing. **Nicole M. Rankin:** writing – review and editing. **Claire Nightingale:** writing – review and editing. **Kate Broun:** writing – review and editing. **Hailey Fisher:** writing – review and editing. **Orelia Bello:** writing – review and editing. **Vivienne Milch:** writing – review and editing. **Rachael H. Dodd:** conceptualisation, methodology, formal analysis, investigation, data curation, supervision, funding acquisition, writing – review and editing.

## Ethics Statement

This study was approved by The University of Sydney Human Research Ethics Committee (2024/HE000700).

## Conflicts of Interest

The authors declare no conflicts of interest.

## Supporting information

Supporting File 1.

Supporting File 2.

Supporting File 3.

Supporting File 4.

Supporting File 5.

## Data Availability

The data that support the findings of this study are available from the corresponding author upon reasonable request.
