# Supplementary material for: User Testing of Information Materials Developed for the Australian National Lung Cancer Screening Program: A Qualitative Study
Source: Health Expect. 2026 Feb 10;29(1):e70592. doi: 10.1111/hex.70592 (PMC12891978; doi:10.1111/hex.70592)
Supplement: Supplementary file 2 — Supporting File 2. [file HEX-29-e70592-s002.docx]

**List of information resources developed for the community** [Available at: <https://www.health.gov.au/our-work/nlcsp/resources/general-public>]

1. Brochure 1 What is NLSCP
2. Brochure 2 Radiology Clinic Visit
3. Brochure 3 Results
4. Brochure 4 Understanding Lung Nodules
5. Brochure 5 Fact Sheet About Lung Cancer
6. Brochure 6 Fact Sheet About Ineligibility
7. Brochure 7 Frequently Asked Questions
8. Scanning Video_Story Board
9. Eligibility Tool
