# Supplementary material for: User Testing of Information Materials Developed for the Australian National Lung Cancer Screening Program: A Qualitative Study
Source: Health Expect. 2026 Feb 10;29(1):e70592. doi: 10.1111/hex.70592 (PMC12891978; doi:10.1111/hex.70592)
Supplement: Supplementary file 3 — Supporting File 3. [file HEX-29-e70592-s004.docx]

**List of information resources developed for the health workforce** [Available at: <https://www.health.gov.au/our-work/nlcsp/resources/health-sector>]

1. Brochure 1 Get your practice ready
2. Brochure 2 Stepwise Flowchart
3. Brochure 3 Key Evidence for NLCSP
4. Brochure 4 Full Resource for GPs
5. Brochure 5 Frequently Asked Questions
6. Brochure 6 Conversation Starters
7. Brochure 7 Pathway Infographic
8. Brochure 8 Support Resources
9. Brochure 9 Talking About Stigma
