# Supplementary material for: User Testing of Information Materials Developed for the Australian National Lung Cancer Screening Program: A Qualitative Study
Source: Health Expect. 2026 Feb 10;29(1):e70592. doi: 10.1111/hex.70592 (PMC12891978; doi:10.1111/hex.70592)
Supplement: Supplementary file 4 — Supporting File 4. [file HEX-29-e70592-s005.docx]

**Supplementary File 4** Interview/focus group guide

Good morning/afternoon/evening. My name is _________________________ from The Daffodil Centre, a joint venture between Cancer Council NSW and The University of Sydney. As part of an engagement with Cancer Australia, we are interviewing people to get their thoughts on information materials developed for the National Lung Cancer Screening Program.

The information we collect will help us better understand how the materials can be used and whether they are helpful. We will then use these findings to edit and adapt the materials and how they can be used for people thinking about taking part in lung cancer screening.

For this interview/focus group, we would like to get your thoughts on the materials and also check that how we have written the tool is easy to understand and helps increase your knowledge and awareness about the National Lung Cancer Screening Program.

You may want to rephrase what you think the text is saying, share how the text makes you feel, share a story that describes something in the text that you’re familiar with, remark on something in the text that is confusing, or say something else that helps you understand the text you’re reading better.

All of the information we collect in this interview/focus group will be kept confidential. The interview/focus group should take no more than 1 hour to complete. I will also be audio and video-recording the interview/focus group so that I can concentrate on your responses and refer back to them during analysis. Is this okay with you?

Do you consent to take part in this interview/focus group? **Ask for Consent Form if we don’t have it***

Do you have any questions before we begin?

**Start recording***

1. What do you think about the materials overall?
2. How easy or hard did you find it to understand the information you read?
3. Tell me about any parts of the materials that you liked
4. Tell me about any parts of the materials that you disliked
5. Do the materials cover all the things you think are important?
6. Is there any information missing that you would have liked to be included?
7. Are there any **other comments** you have about the materials, or anything else we’ve not touched on?

For community members interview ONLY: Section by section (teach-back), ask interviewee to say in their own words what they understand each section to mean.

*Thank you again for your time. I wish to remind you that all the information you share with me today will be kept confidential.*
