# Supplementary material for: User Testing of Information Materials Developed for the Australian National Lung Cancer Screening Program: A Qualitative Study
Source: Health Expect. 2026 Feb 10;29(1):e70592. doi: 10.1111/hex.70592 (PMC12891978; doi:10.1111/hex.70592)
Supplement: Supplementary file 5 — Supporting File 5. [file HEX-29-e70592-s001.docx]

**Supplementary File 5** Summary of key findings (shared/distinct themes) with illustrative quotes

| **Category** | **Theme** | | **Illustrative quotes** | |
| --- | --- | --- | --- | --- |
|  | **Shared theme** | **Distinct theme** | **Community** | **Healthcare professional** |
| **Understandability** | Language found to be clear and easily understandable |  | - 1. I'm suffering from everybody that did smoke. And have a history of at least 30 pack years of cigarette smoke. OK, yes, that is pretty self-explanatory. (CM-1)**^1^**   2. I mean, it's very clear and I think. Probably better than a lot of the materials I've seen put out for education programmes… I think that the process is very clear...I just think it's easy to understand. It's very explanatory and you know, it just makes it clear. (CM-2)   3. it’s informative explains it in simple words for everyone to understand. (CM-3)   4. I thought overall they were very clear and easy to understand. (CM-4)   5. I love the fact that it's actually use a simple language...for like different people it's very easy to understand (CM-5)   6. I think it's very good overall...Most of it's clear, it's easy to understand. (CM-13)   7. The majority of it is clear and easy to understand. (CM-14)   8. I actually thought that they were all very clear and easy to understand and they sort of followed on well. (CM-18)   9. ...to me it's fairly self explanatory. (CM-24)   10. It's very clear to understand. (CM-25) | - 1. I like the document, thought it was clear. (HW-FG-1)**^2^**   2. The information that when you actually read it is very clear. (HW-I-3)^3^   3. … reading through them [information resources], like I think they've been developed really well. (HW-I-9) |
|  |  | CM: Need for clarification of some professional terms and concepts | 1. The last one's a bit. Confusing. Have a history of it, 30 pack years. Do they mean 30 a day or a pack? ….Getting people as an ex smoker, and one who's lied to doctors blatantly. ...it'd be very hard to get the truth out of the smoker for that. (CM-3) |  |
|  |  |  | 1. I'm not too familiar with radiation and that sort of stuff and the effects that will have on the body,...you need to back it up with something... You almost gotta just explain what is [natural] radiation, where it comes from...(CM-6) 2. The term pack years might be the only slightly negative thing with that in that I'm not sure everybody would know what a pack was. (CM-9) 3. I guess the low dose means the radiation...I know about the radiation, but other people may not...and people are a bit worried about radiation. See this is have your low dose CT scan, so maybe in brackets low dose radiation. (CM-10) 4. …they [people who smoke] are smoking so many other things…methamphetamine and all those… I did smoke cannabis, they [people who smoke] would put some threads of tobacco in to make it stay burning properly. So yeah, my pack is, it's very difficult to define, so I don't know how you would. I mean somebody who's just like my brother, who's just been smoking cigarettes. He's probably busy to work out, but I don't know how I would work it out... (CM-11) 5. ...some people might not even be aware that there is natural radiation... Why don't you say lower than one year exposure to naturally occurring radiation. such as whatever it is, sunshine or whatever...(CM-12) 6. I don't know how to calculate that [pack-year]. That's the only thing that comes to mind. And this really doesn't tell me. Yeah. How do I work out that number myself? (CM-14) 7. I had an issue with that [pack year]. (CM-16) |  |
|  |  |  | 1. what is natural radiation then? Like from from a layman's understanding, what's happening?...I don't know, I don't get exposed to radiation. They they wouldn't have no idea what, where those sources, natural sources of radiation comes from. I think I think and I. I'm just being mindful that not everyone has that level of understanding...Maybe in brackets behind natural radiation, just give a few examples of what that includes...whether it be exposure to mobile phones or other sources of radiation. (CM-17) 2. [provide some examples of natural radiation?] I think so... I suppose other people [don't know the natural radiation]. Well, is it just from the sun? Or is it exposure to other things like?...maybe some examples there. (CM-19) 3. [is it helpful to explain what is a pack year?] I think so because that's not clear. (CM-23) 4. I've had a chest CT and that nothing was ever said about low dose or. And So what does the low dose mean? (CM-24) |  |
|  |  | HW: Need for concise use of professional nomenclature and concepts |  | 1. An authorised medical practitioner. Other times you're talking about GPS. So who's an authorised medical practitioner? (HW-FG-1) 2. Who are the other medical practitioners? (HW-FG-2) 3. Did you switch [cigarette smoking] to rollies? (HW-FG-5) 4. How can my patient self-refer? How does that like no one can self-refer? (HW-FG-7) |
|  |  |  |  | 1. How can my patients self-refer for lung cancer screening? (HW-I-1) 2. Tobacco…[how about] shisha smoking? (HW-I-5)   And I thought, oh, does that mean like, if you haven't done the or courses, then you're not an authorized medical practitioner to be doing the screening yet? So yeah, that [authorised medical practitioners] that's a very confusing word. (HW-I-7)   1. Self-referral is unclear. (HW-I-9) 2. What if my patient smoked cigars, pipes, vapes… It just says the best evidence is support cigarette smoking. (HW-I-10) 3. What about chewing tobacco?. (HW-I-11) 4. But how can a patient self-refer?. (HW-I-12) 5. Self-referral wording is unclear. (HW-I-14) |
|  |  | HW**:** Need for clarification of process details in some areas |  | 1. …Who gets registered for NCSR - clinic or individual GP? Not clear…Where do we send patients for scan and follow up/nodules?... How do results get to GPs, make clear - notification to inbox? PRODA? - If PRODA then this is additional work (HW-FG-3) 2. Will reminders be sent by NCSR? Make clearer…Clarity about recall and who's responsible for follow |
|  |  |  |  | up scans. (HW-FG-2)   1. Who is responsible for recall of investigation? Who calls patients? (HW-I-15) 2. Follow up scan is unclear. Does this mean LDCT or other scans/tests as well? (HW-I-8) 3. What happens if patient changes |
|  |  |  |  | practice? How to know if they are enrolled? (HW-I-3) |
| **Usefulness** | Information found to be comprehensive |  | - 1. …it covers everything that I I could imagine it needing to and even, you know, like you said, what to do if you don't qualify for the programme. (CM-2)   2. It's covering [everything], like you've got your, your main information, what you got basically got your titles. What do I need to do and it kind of it's like a step through process. (CM-6)   3. I think they're all covered. What is lung cancer? What is the screening? Why should I consider? What are the potential concerns? Well, radiology clinic. What happens next? I think that's good. (CM-9)   4. …second page information helpful. (CM-15)   5. I've found it very comprehensive reading the whole package. (CM-17)   6. that's quite easy to understand. Yeah, quite comprehensive, covered everything. (CM-21) | - 1. [Key evidence] Useful information. (HW-FG-2)   2. Comprehensive, salient points covered…for a new program [Full resource for GPs] is useful…[Stepwise] document is most useful, could be central document with links to all other docs…Things to remember section [in the Checklist Get Ready] not helpful. (HW-FG-3)   3. Not useful page on back [of the Checklist Get Ready]. (HW-FG-7)   4. [Full resources for GPs] helpful. (HW-I-13, HW-I-2)   5. Key evidence] Useful… Results info useful…[FAQs] All useful. (HW-I-5)   6. Evidence page very useful. (HW-I-11)   7. Things to remember [in the Checklist Get Ready] isn't useful…[FAQs] helpful (HW-I-13) |
|  |  | CM: Enhancement of practical value for the community | 1. And then I think there needs to be a greater emphasis on encouraging, like getting younger people to encourage their older family members to get screening. Yeah. I feel like that's a huge issue...It's not emphasised enough in these documents I feel...Yeah, I think it should be included in the FAQ as well to encourage younger people to get their older family members to get screening done. (CM-13) 2. …I don't know if there should be comments [in the scanning video] like if you're part of the |  |
|  |  |  | member who you think fits eligibility criteria they should encourage them to get screening. (CM-25) |  |
|  |  | HW: Enhancement of practical value for the health workforce |  | 1. There'll be certainly people I would see here who have been regularly smoking bongs for 30 years, and I figured that that's got tobacco in it. So some guidance on on the bong equivalent of of a pack year will help. (HW-FG-1) 2. Was there a statistic about like how much that [grams per week for rolled cigarettes] is equivalent to [pack-years]… I haven't found any equivalent packages for that. (HW-FG-2) 3. There should be some kind of, you know, explanation or readymade scripts that we can give to patients back up our position, why that person did not get through the eligibility…. who are worried about their risks, |
|  |  |  |  | especially if their family members or their relatives or coworker had lung cancer, and they are excluded from these screening program. So what do we do in those group?... how we can consult patients in that in that regard. (HW-FG-3)   1. [Need] calculator for pack history. (HW-FG-6)   And I guess to the other thing is that you've got about calculating smoking history, but is it worth pointing people to tools where they can calculate? (HW-FG-7) |
| **Usability** |  | CM: Adoption of community-friendly design | 1. What I liked about it [the grade of risk] was it sort of it put down, you know, the various levels of concern, if you like, That makes it very clear that you know number one is very low risk, not no risk…. I just like the fact that it goes all the way up to [level] four and five. To me, it's sort of, you know, gradual scale if you like and it's not alarmist though. (CM-4) 2. …so I feel like these symptoms include a cough that doesn't go away coughing up blood. It should be actually probably in bold letters or like somebody. (CM-5) |  |
|  |  |  | 1. I like the little eligibility kind of little icons there. So that's that's really catching and it strikes the point…I think this is really good…You got little tick boxes. I think that's great…if you got like a little step by guide. And then it just just takes away some of the the stress of not knowing what   you're about to head into. So I think that's really |  |
|  |  |  | good. (CM-6)   1. It's not too much. It's simple…. I can imagine myself going, oh OK I'll tick off 1 and OK. Yes, the doctor. OK find out where it is. I think that's a good heads up for everybody. (CM-8) 2. I found it all very good because it's breaking the whole thing [lung cancer screening program] down into digestible bites...I like this (eligibility figure). I think I think it's very straightforward. I think it's very clear. (CM-9) 3. And the pictures are quite, you know, helps you to understand it more. (CM-10)   I like the green coloured graphic [eligibility |  |
|  |  |  | criteria] on the left-hand side cause that condenses it [the complex information] into [a figure], easy to read I think...So I started reading all the texts before I saw the green bits. If I'd seen the green bits first, I would have gone straight to that cause it's quicker and easier to understand. (CM-12)   1. [Page 1] maybe there's some big text you know below that [photo]. (CM-21) 2. It (eligibility figure) Is very clear to understand there's like images with very few words, so it's like very concise. It's not hard to read and it looks good. Like the colour is actually flowing really well. (CM-25) |  |
|  |  | HW: Adoption of health workforce-friendly design |  | - 1. likes checklist [Checklist Get Ready], answers many of questions. (HW-FG-1)   2. [Use] pictures and graphs for some of the evidence. (HW-FG-2)   3. lots of information, need to read many times… Font too small, text |
|  |  |  |  | - 1. heavy, can be shortened (HW-FG-3)   2. Small print and lots of words. (HW-FG-4)   3. If it's presented in tables with the reference below, it would save some space and visually will be easier to read than going through the whole lines. (HW-FG-5)   4. …want 1-2 page document only. (HW-I-4)   It's easier to read the last form that you showed me than this |
|  |  |  |  | one. But it's the same. It's the same information. (HW-I-10)   - 1. I like the graphics on it…The themes coming through with all the fonts and the colours which keeps everything quite consistent. The eye goes to what you need to read. (HW-I-12)   2. Looks slightly overwhelming. (HW-I-13)   3. GPs like quick and succinct. (HW-I-14) |
| **Desirability** | Appreciation of visually engaging design |  | 1. I'm always attracted or interested in seeing someone, what I call a normal person or a picture of a normal person saying ‘I never knew I had whatever it was, I'm so happy I got there and these are nice coloured pictures and they look welcoming. You know, that something you want to look at. (CM-2) 2. The pictures that involve, the images actually pleasing to the eye because it makes me want to | 1. [Conversation starters] Looks good, eye catching (HW-I-12) |
|  |  |  | 1. know what this whole thing is about. It makes me want to try. (CM-5) 2. I really like the logo… it's an easy, appealing thing to look at, like before, I was sitting somewhere and I had a couple of minutes where I had nothing to do. I'd probably pick up and read it, cause it looks appealing. It's got a nice soft, approachable tone to it. So. Yeah, usually a lot of the Australian government stuff, very dry and kind of harsh, but looking at this, I think it's looks really good. (CM-6) 3. And I like the imagery here. It's very reassuring. I mean, it's a frightening thing to go for screening, |  |
|  |  |  | right, because you know you have this optimism bias, it's going to be OK. Final slide- this doctor's face is very reassuring and comforting. (CM-7)   1. …and the pictures of the people make it humanising as well. (CM-9) 2. To pick up an actual brochure, I have to feel I have to feel that hook. I have to be reeled in like a fish. So yeah, the the front cover of it's critical. Calm blue. Probably brings thoughts of medical clinical da da da it needs to be warm up. Please come in and have a look at me because you really need to. Yeah, OK, bright yellow. Yeah, that sells. (CM-11) 3. I prefer real people, real people and put a picture of the gown as well, maybe hanging on the wall...I want to see real people in the video because this one. It's not very clear with images. Real people are more effective... everything to be real images. (CM-20) |  |
| **Accessibility** | Need for diverse |  | 1. I like that easy to read format [A4 version]...that one does appeal to me. I find it easy to read… I | 1. [Add] clickable table of contents for online version. (HW-FG-1) |
|  | information access channels |  | quite like the first one actually [A4 version]...all the writing and the question that one was good...Less busy, less busy, and I don't know, maybe that's just my style of. (CM-2) | 1. Would rather portrait A4 document. (HW-I-13) 2. Would prefer A4, do we need A3? (HW-I-14) |
|  |  |  | 1. I think it's important to have it in the audio form. (CM-4)   I think a combination of approaches (dynamic and static information) works very well because you're not reading the same format all the time, so the brain is processing it differently…I think the video concept is good because people have different learning and communications styles |  |
|  |  |  | and it's nice to give a variety of approaches. (CM-9)   1. Because if it's both [presented online and in the pamphlet], when I first saw it, I thought ohh, do I need to click or I just follow it down and it answered its own question. So it that worked really well. Yeah, having an arrow to the right...that's really a digital indication…if it's print, you might need a different icon. (CM-11) 2. I like the trifold just because when I receive trifolds, I feel like it's more. It's, it's synonymous with the information that you get in the GP clinics. (CM-17) |  |
|  | Need for content inclusivity |  | - 1. I feel it lacked a bit of cultural diversity that's probably my main comment. I suspect that the groups that are harder to reach probably might be more culturally diverse, particularly won't read all of the wording in this. So even the doctors and the healthcare team - they're so very white as well. What do I need to do? I think the wordiness of the information about what you need to do. Perhaps it could be done in a | - 1. What does it [smoking] mean in different cultures? … How is smoking perceived and that kind of stuff. (HW-FG-7)   Probably really not helpful. So. Like, there's already training involved in the fellowship of because it doesn't direct us to any resources about what other |
|  |  |  | picture pictogram as well. There will be people who read it, but I don't think you'll get the majority of people reading all of the information in the pamphlet. (CM-7)   - 1. [At your scan] Is there an interpreter service? (CM-16)   2. [Special arrangements] Some people just can't walk into a clinic to get a test...they're in a wheelchair. Any special arrangements to help you attend...If you need an interpreter. (CM-19)   Also add for more information in other languages, please go to the website or ring TIS on number. (CM-20) | deceptions and what does it [smoking] mean about different cultures. (HW-I-1)  2.2 Could add cultural aspects from main GP resource. (HW-I-5) |
|  |  | CM: Need for language inclusivity | - 1. I also jotted down would it be available in other languages? …I assume this would be available in other languages for those for whom English is not their first language? (CM-4)   2. I like how the language is written in a way that's accessible for everyone. It's not technical; it's not medical jargon. It's plain language plain English. (CM-17)   3. I notice you have something written in the bottom right hand corner that is written in another language and it made me wonder what is happening about getting this available for non English speaking peoples. (CM-24) |  |
| **Credibility** | Appreciation of authoritative branding and logo |  | 1. I really like the logo, national lung cancer screening programme. So the blue and the green and they they're the two key colours that. That appear throughout the document, so I think that's really good…it looks like a lung and it's blue and green… Blue air, maybe. I'm not sure, but the green is kind of soft and, you know, it's an easy. Appealing thing to look at…usually a lot | 1. But the National [Asthma] Australia or something have a very similar colour palette and lungs, which I'm not saying is a bad thing because they both got lung things, but the colours are very similar… it's got a very similar blue-green sort of colour. I |
|  |  |  | kind of harsh, but looking at this, I think it's looks really good. (CM-6) | doubt it will get mixed up…but I quite liked it. (HW-FG-1) |
|  | Appreciation of transparent communication |  | 1. Some of them are slightly wordier than others, but they're, you know, honest about what is lung cancer. (CM-4) | - 1. Suggest acknowledging unknowns of program, in order to build transparency and trust. (HW-I-15) |
| **Affiliation** |  | CM: Value information pertinent to personal health experience | - 1. What I did find interesting was that as somebody who has had a lung nodule diagnosed many years ago... I knew that it was a potential for problems, but I never quite put together with lung cancer...it became pertinent to me and relevant. (CM-4)   2. I like the second page, particularly number 5 |  |
|  |  |  | I think people realised that, but in my case. I was having heart scans and they picked up issues in my lungs. So same thing. Yeah, that's fine…I think it's pretty clearly set out...[have covered everything you want to know?], yes I think so. (CM-12) |  |
|  |  | HW: Need for building connections with professional roles |  | 1. that was an 8 page document intended for health practitioners…Not necessarily a contents page, but what this document is, who is it for… (HW-FG-6) 2. guess like. For me, that's not so relevant. I think that more has to be on like a practice level. It's about making sure that the right people see the right like resources. Or what information is for who in this. (HW-I-13) |
| **Findability** | Not evaluated | | | |

^1^CM-X refers to community member X; ^2^HW-FG-X refers to health workforce’ focus group X, ^3^HW-I-X refers to health workforce ‘individual interview X.
